# Parents’ motivations, concerns and understanding of genome sequencing: a qualitative interview study

**DOI:** 10.1038/s41431-020-0575-2

**Published:** 2020-01-30

**Authors:** Celine Lewis, Saskia Sanderson, Melissa Hill, Chris Patch, Beverly Searle, Amy Hunter, Lyn S. Chitty

**Affiliations:** 1 London North Genomic Laboratory Hub, Great Ormond Street Hospital for Children NHS Foundation Trust, London, UK; 2 UCL Great Ormond Street Institute of Child Health, London, UK; 3 Institute of Health Informatics, University College London, London, UK; 4 Genomics England, Queen Mary University of London, Dawson Hall, London, UK; 5 Faculty of Health and Wellbeing, Sheffield Hallam University, Sheffield, UK; 6 Society and Ethics Research, Wellcome Genome Campus, Cambridge, UK; 7 Unique—Rare Chromosome Disorder Support Group, Oxted, UK; 8 Genetic Alliance UK, London, UK

**Keywords:** Human behaviour, Social sciences

## Abstract

The 100,000 Genomes Project is a hybrid clinical and research project in which patients and parents are offered genome sequencing for cancer and rare and inherited disease diagnosis; all participants receive their main findings and contribute their data for research, and are offered optional secondary findings. Our aim was to explore participating parents’ attitudes towards and understanding of genome sequencing in this hybrid context. We conducted in-depth telephone interviews with 20 parents of children with rare diseases participating in the 100,000 Genomes Project. Parents were positive about contributing to research, although some had needed reassurance about data protections. Although most felt positive about secondary findings, some could not recall or misunderstood key aspects. Some were also concerned about potential emotional impact of results and a few raised concerns about life insurance implications, and the impact of future legal changes. Participants were generally positive about consent appointments, but several raised concerns about ‘information overload’ because of deciding about secondary findings at the same time as about the main diagnostic genome sequencing and data contribution. Additional information resources, particularly online tools, were highlighted as potentially useful ways of supporting the consent process. We conclude that parents offered genome sequencing as part of a national hybrid clinical and research project report many positive attitudes and experiences, but also concerns and misunderstandings. Further research is needed on how best to support informed consent, particularly about secondary findings. Additional resources such as online tools might usefully support future genome sequencing consent processes.

## Introduction

Next-generation sequencing technologies including genome sequencing are increasingly being utilised to aid the diagnosis of children and adults with rare and inherited diseases [1, 2]. The search for a genetic diagnosis can be a long and arduous journey, and patients are likely to have undergone extensive medical testing during this time [3]. Genome sequencing has the potential to reduce this ‘diagnostic odyssey’ for many patients and their families. The benefits of obtaining a genetic diagnosis include the potential for more accurate treatments, a clearer prognosis, information about recurrence risk and opportunities to make contact with other families with the same diagnosis through support groups [4, 5]. The diagnostic yield of genome sequencing for previously unsolved paediatric cases is up to around 40% (depending on the clinical indication), and this is likely to continue to increase as knowledge grows [1]. In addition to the benefits of genome sequencing, there are also potential challenges including: identifying patients’ information needs, facilitating decisions about testing and research participation, managing varying levels of genomic literacy, managing patients’ expectations and determining whether and how secondary findings should be offered to patients [6, 7].

The 100,000 Genomes Project was set up in England in 2015 to pilot the introduction of genome sequencing as part of a new NHS Genomic Medicine Service. The 100,000 Genomes Project is a hybrid clinical practice and research project, with four overarching aims: “to bring benefit to patients and set up a genomic medicine service for the NHS; to create an ethical and transparent programme based on consent; to enable new scientific discovery and medical insights; and to kick-start the development of a UK genomics industry” [8, 9]. Tens of thousands of patients with cancer, or with selected rare and inherited diseases, and parents of the patients with rare diseases, were enrolled between 2015 and 2018. Receiving main findings relating to the proband’s condition, and having sequencing data and medical records stored in a biorepository for research purposes, were conditions of participation. Participants could also choose whether they wanted to receive clinically actionable ‘secondary findings’ (or ‘additional findings’) in their DNA sequence data. The genes/conditions currently on this secondary findings list (although subject to change) include hereditary breast and ovarian cancer (*BRCA1/2*), hereditary colorectal cancer (Lynch syndrome) and familial hypercholesterolaemia, as well as parental carrier status for cystic fibrosis [10]. These are not labelled ‘incidental findings’ because they are actively searched for rather than arising unexpectedly. At the time of writing (15^th^ August 2019), no secondary findings had yet been returned to participants.

The subject of secondary findings raises a number of ethical and clinical issues. Whilst studies have shown that most key stakeholders think that both actionable and non-actionable secondary findings should be returned [11, 12], healthcare professionals have acknowledged the increase in workload of clinical scientists and clinicians in interpreting and returning such results [12]. Questions have also been raised about who should consent patients and deliver test results [13]. The potential to cause anxiety or psychological harm to patients associated with ‘the burden of knowing’, as well as concerns around discrimination in insurance and employment, have also been identified [12].

As genome sequencing is now being implemented in healthcare systems globally, including in the new NHS Genomic Medicine Service in England, it is important that patients’ views and experiences of genome sequencing are understood. In-depth qualitative research can provide insights on patients’ views and experiences that may be valuable to policy makers, healthcare professionals and researchers involved in developing resources, information materials and protocols for future genomic medicine projects and services. For example, interviews with patients can highlight topics covered during the consent process that were less well understood, identify patients’ information needs and uncover patients’ concerns that need addressing. In addition, emergent themes from this type of in-depth qualitative research can be used to generate testable hypotheses and develop new measures for use in large-scale quantitative survey studies [14].

A few recent qualitative interview studies have started to shed light on the perspectives of parents who have been offered genomic (exome or genome) sequencing to diagnose their child’s rare conditions in the UK [15], US [16, 17], Canada [18] and the Netherlands [19]. These studies have highlighted common motivational factors for consenting to genomic testing such as desire for a diagnosis and contributing to science [16], but have also identified concerns around elevated expectations and loss of hope for recovery following a diagnosis [17]. The 100,000 Genomes Project now provides a valuable opportunity to learn how patients and their relatives respond when offered genome sequencing within the UK NHS, in a hybrid clinical and research context, including mandatory contribution of personal health data for research, and optional secondary findings. One previous qualitative interview study with 20 participants in the 100,000 Genomes Project was recently published [20]. That study highlighted participants’ attitudes specifically regarding trust in the NHS and views towards data access by commercial companies. However, participants’ broader attitudes towards, and understanding of, the diagnostic and secondary findings offered were not explored.

Our overarching objectives in this study were therefore to explore the themes that arise when participants talk about their understanding, attitudes and experiences regarding the clinical diagnostic aspects, contributing personal health data for research purposes and secondary findings when undergoing genome sequencing in this clinical and research context. Our primary aim was to identify themes that would be useful for policy and practice, particularly for the new NHS Genomic Medicine Service and other similar healthcare systems now being implemented. Our secondary aim was to use emergent themes to develop new quantitative survey measures for use in ongoing and future large-scale genomics research on patient-reported outcomes and experiences.

## Methods

### Study design and setting

This was a qualitative interview study conducted after parents had consented to genome sequencing, but before they had received any main or secondary findings from the project. Approval was obtained from the NHS Research Ethics Committee West Midlands (15/WM/0258). Recruitment was conducted in a London children’s hospital in a Genomic Medicine Centre between June 2016 and March 2017.

### Participants and recruitment

Participants were eligible if they were: parents of children with rare diseases who had consented to genome sequencing in the 100,000 Genomes Project and had blood taken; were able to read the information materials and could complete the interview in English. To recruit participants for the interviews, healthcare professionals who were consenting families to the 100,000 Genomes Project invited a continuous sample of potential participants at the end of the consent appointment. After being given a short description of the study, parents who were interested were asked to complete a ‘consent to contact’ form with their name and contact details. One of two researchers (SS, CL) then contacted interested parents to discuss the study further with them; interested parents were then sent a participant information sheet via email or post. A telephone interview was then arranged with those willing to take part. Parents of children with rare diseases often have multiple caring commitments and children with complex needs, and many of the families attending the recruiting hospital live far away. We therefore opted for telephone interviews rather than face-to-face to make it more convenient for the parents.

In total, 57 parents were initially approached to take part in this study and signed a ‘consent to contact’ form. Attempts were made to contact the first 40 of these parents. Of these 40, 14 were ‘passive decliners’ (six did not answer the phone calls and eight did not reply to emails) and six were ‘active decliners’ (five agreed to participate but subsequently did not answer when called for a scheduled interview, and one actively declined to participate when invited). Data saturation was reached after 20 interviews. The remaining 17 parents who had initially been approached were therefore not contacted. The 20 interviews were conducted between 5 days and 17 weeks following recruitment into the 100,000 Genomes Project (median 7 weeks). The mean interview length was 28 min, and interviews ranged from 11 to 53 min. The 11-min interview was an outlier and was particularly brief because the participant gave only very short answers to all questions despite the open-ended questions and probes. Sixteen of the interviews were conducted by SS and four by CL.

#### Topic guide

An interview topic guide (Supplementary File) informed by the literature [21] and the advisory team (comprising three genetic health professionals, two social/behavioural scientists, two patient advocates from support groups and a lay member who was a participant in the 100,000 Genomes Project), was developed by SS and CL with questions being refined after the first few interviews had taken place. Topics included: understanding (the purpose of genome sequencing, whether they will get a result, how their genomic data will be used, who will have access to it etc); motivations and concerns around main findings and secondary findings including whether they consented to secondary findings; information needs and satisfaction with the consent procedures and materials.

### Data analysis

Audio recordings were transcribed verbatim and de-identified. Thematic analysis was used to analyse the transcripts, which involved an iterative process where data were coded, compared, contrasted and refined to generate emergent themes [22]. The themes were organised to map on to the overarching aims, that is, exploring understanding and attitudes towards diagnosis, research uses of data and secondary findings. To develop the code book, two investigators experienced in qualitative analysis (SS and CL) each read three transcripts and went through an iterative process of revising and refining the code book. First, SS and CL independently developed an initial draught codebook informed by the aims of the study and by reading and reviewing the same transcript; these were compared and combined into a single codebook after discussion. Next, the two investigators independently coded a second transcript; disagreements were resolved, and minor codebook revisions were made. Each investigator then independently coded a third transcript. The Kappa for the two sets of codes indicated good inter-rater agreement. Both investigators then independently coded the remaining transcripts using the final code book (SS coded nine and CL coded eight). Once all transcripts were coded, both investigators reviewed and revised the codes together. NVivo 10 (QSR International, Australia) was used to manage the data and facilitate coding. Names used in quotes are not individuals’ real names.

## Results

### Participants

The 20 participants were parents (13 mothers, 7 fathers) of 21 children affected by a range of rare conditions (Table 1), of whom eight had no diagnosis and 13 had a general ‘working diagnosis’ (i.e. a broad label used to describe the condition e.g. intellectual disability, autism or epilepsy) with an unknown genetic aetiology.

**Table 1:** Participant characteristics.

Characteristic	*N* or range and mean
Age of participant	25–49 years; mean = 39 years
Sex of participant	
Female	13
Male	7
Age of child	10 months–18 years; mean = 10 years; 2 children were deceased
Gender of child	
Female	8
Male	13
Condition type	
Neurological	6
Intellectual disability/ developmental delay	5
Metabolic	3
Mitochondrial	2
Endocrine	2
Cardiovascular	1
Dermatological	1
Primary ciliary disorder	1
Diagnosis with unknown genetic aetiology	
‘Working diagnosis’	13
None	8

One parent had two children enrolled in the study.

### Attitudes towards the clinical purpose of the sequencing

#### Understanding

Participants understood that a major reason for their being invited in to the 100,000 Genomes Project was to obtain a diagnosis for their child, and several evidenced understanding that this could have potential implications for family members. Around half explicitly stated that they might not get a diagnosis. See Table 2 for illustrative quotes.

**Table 2:** Understanding and attitudes towards the clinical purpose of the sequencing.

Categories	Themes	Example quotes
Understanding	To obtain a diagnosis	*“This is where they can do testing for him to try and find his diagnosis” P07*
	Implications for family members	*“Sarah has got two brothers…if they’re carriers of the gene then they may pass that on” P02*
	Might not get a diagnosis	*“we’re very realistic about the potential for it to come back with nothing” P01*
Perceived benefits	Desire for diagnosis	*“when you spend so much time trying to find a diagnosis, you’re not going to say no” P18*
	Last opportunity for diagnosis	*“If this can’t yield [a diagnosis], I don’t know else would to be quite honest with you” P06*
	Desire for clearer prognosis	*“for information potentially about life expectancy” P04*
	Need to understand why	*“That need to know never leaves you… you always wonder why, how, was it you, was there something you could do?” P16*
	Possibility of better treatments	*“develop a drug that’s not an injection” P15*
	Family planning	*“If [my daughter] is going to have children, is it going to be passed on?” P16*
	Curiosity about the rare condition	*“wouldn’t impact treatment because they already know what’s going on” P15*
Concerns	Prognosis more serious than initially anticipated	*“if it’s one of the syndromes then there’s a possibility he could go blind” P02*
	Anxious might have passed condition on	*“it’s also hard because if something comes back in my DNA that says that I cause my son to be the way he is…that’s going to be really hard to accept” P07*
	Not wanting to get your hopes up	*“”I don’t want to get my hopes up that there is a definite diagnosis that we’re going to receive” P16*
	Blood test	*“[my child] doesn’t like blood tests” P08*

#### Perceived benefits

All participants stated that their desire for a diagnosis was an important reason for their agreeing to genome sequencing. They wanted a *“definite answer”*, to *“get to the root of the problem”* or, where there was already a diagnosis based on clinical features, to obtain a better understanding of the underlying genetic cause of their child’s rare condition. Several referred to genome sequencing as being the last opportunity to get a diagnosis, e.g. it was something *“we’ve got to do”*. Some talked about wanting a diagnosis in order to have a clearer prognosis for their child, e.g. *“to know what his future holds”*. A few talked about the need to understand why their child had died and the emotional impact of not knowing (*“having all of that weight on your own shoulders”*). A number of participants discussed the possibility of improved or more accurate treatments for their child, e.g. *“develop new medication.”* Only a couple of participants mentioned *“possibly finding a cure”* for their child. Several parents talked about being generally curious about the rare condition rather than anticipating specific benefits. The other key benefit was around family planning, either for themselves or their children.

#### Concerns

A number of participants expressed concerns about the potential emotional impact of receiving, or not receiving, a diagnostic result from the genome sequencing. For example, a few were anxious that the diagnosis might show the prognosis to be more serious than anticipated, with one commenting that a diagnosis of one particular condition would confirm the possibility that their child could go blind. Within this theme, some expressed concerns about how they might feel if they found out they had passed on the condition to their child, for example, *“that’s going to be really hard to accept”*, and a few spoke of not wanting to get their *“hopes up”* in case they did not get an informative result. The only practical concern cited was the child’s dislike of blood tests.

### Attitudes towards the research uses of their data

#### Understanding

Participants appeared to understand that, in addition to the clinical diagnostic purpose, a major goal of the 100,000 Genomes Project was to contribute to research (*“wider research”* and *“advance medicine”*). In terms of demonstrating understanding of who would have access to their data, there was evidence in several interviews that participants understood commercial organisations would be allowed to access their child’s genomic data, but only one participant explicitly stated university researchers could also potentially access the data. In terms of demonstrating understanding how their data would be protected (data protection), several reflected understanding that the *“information is anonymised”* and *“the database will only hold ID numbers and no names”*. Several also displayed understanding about how the data would be stored (data storage), e.g. that it would be held in a *“national database”*. However, one mistakenly thought the database would be kept within their specific healthcare institution. None of the participants specifically exhibited the understanding that, because rare diseases can affect extremely small numbers in the UK, there was a small risk that they or their child might be identified as someone taking part in the project.

#### Perceived benefits

When asked about their motivations for genome sequencing, around half the participants said that they were motivated by a desire to help others with the same condition via the research aspect of the 100,00 Genomes Project. Several said they had no concerns even if their genomic data were to be made publically available, if it could potentially lead to beneficial research. Within this theme, a sub-theme was that most hoped their research contribution might eventually lead to a cure for others. A few also commented that by taking part in the research, other children with similar symptoms might be diagnosed earlier. Around half also talked about how their participation might contribute to scientific research more broadly, and to helping *“people in the future”* more generally. Two specifically mentioned wanting to support the NHS or their particular hospital *“any way I can*”. Some talked about this being *“an opportunity [to be a] part [of] moving forward with medicine”*. Some explicitly expressed positive views about pharmaceutical companies having access to their data for research and developing new treatments. A few described participating in the project as *“not a huge commitment”* and *“just a simple thing”* that only required *“giving a bit of blood”*.

#### Concerns

Around a quarter of participants said they had had concerns about data protection and privacy prior to the consent appointment. One of these participants’ concern about data protection was unresolved (ongoing concerns about privacy/security changes); he remained concerned because he had been told that participants would not be informed if any changes were made regarding data access or protections, likening this to *“your bank changing your terms and conditions and never telling you.”*

However, most participants were reassured about their data security during the consent appointment conversation. Reassuring factors discussed included that data were *“going to be anonymised”* or *“won’t be identifiable”*, and that there were legal protections to prevent mishandling of the data, e.g. *“I was reassured [by the consenter] that… if the researcher does use someone’s data illegally then all their funding…would be pulled.”* No concerns were raised about the NHS or academic researchers accessing their data, and only one participant expressed very mild concern about pharmaceutical companies having access, saying: *“I guess concern is not the right word, but…I might have paused”*. See Table 3 for additional illustrative quotes.

**Table 3:** Understanding and attitudes towards the research uses of the data.

Categories	Themes	Example quotes
Understanding	Contribute to research	*“they spoke to us about the consent for the pharmaceutical companies and I think that that’s good because if they’re able to find cures or advance medicine then I’m happy to do it” P02*
	Commercial organisations accessing data	*“I’m well aware you know, companies around the world, private companies, pharmaceutical companies…there’s many companies involved in how research takes place” P19*
	University researchers accessing data	*“you know, if I was against universities looking and doing their research, because after all they get funding…to do that” P06*
	Data protection	*“everything is kept confidential…our names are not matched to the sample” P03*
	Data storage	*“I believe the results will be fed into some kind of central database” P06*
Perceived benefits	Desire to help others with same condition	*“If in ten years’ time if another child is born and the’ve got a similar thing to Jamie…you know?”P07*
	May eventually lead to a cure for others	*“If one day they can find a cure and help other children as well, it will be worth it” P17*
	Help others get diagnosed earlier	*“let’s say another kid comes in with exactly the same display of symptoms and problems as our son, he might get an answer at the age of one or before” P19*
	Support NHS	*“but if we can give something back to the NHS via this new, fledgling project and help you build, you know, another arm o d the NHS through this whole genome sequencing…” P14*
	Importance of pharmaceutical companies having access to data	*“We need to have the pharmaceutical companies to be able to create the medications to try and help with these conditions.” P16*
Concerns	Data protection and privacy	*“So that was my major concern. You don’t want commercial people to be able to identify you. But I think they relayed all – got rid of all those concerns for us.” P18*
	Ongoing concerns about privacy / security changes	*“What happens if in 15 years’ time the rules change in the NHS or the government changes the rules, would you let us know that effectively the contract between us has changed slightly?…I remember I specifically asked ‘will you tell us if things change?’ and she said ‘no’…That’s the point where I thought, hmmm, you might need to clarify that one for the future…. [It’s like] your bank changing your terms and conditions and never telling you.” P19*
	Pharmaceutical companies	*“I guess concern is not the right word, but…I might have paused”. P20*

### Attitudes towards secondary findings

#### Understanding

Most participants recalled unprompted the conversation about health-related secondary findings, although one could not recall and another could only vaguely recall the conversation. Of those who remembered the secondary findings conversation, many participants specifically recalled that additional findings related to cancer could be looked for, and several specifically recalled heart disease. However, a few had misunderstandings about results types, i.e. which secondary findings would be looked for, with some thinking they would get findings about diabetes, Huntington’s disease or sickle cell disease. There were also misunderstandings around the level of control participants had about the process, e.g. one couple thought they could specify which conditions they wanted returned, rather than opting in or out to a set list of conditions. Around one third of participants specifically mentioned carrier secondary findings with only a few of these mentioning cystic fibrosis. Notably, several could not recall being asked and several could not recall their decision if they had subsequently signed up to receive secondary findings (the time since the consent appointment for these participants ranged from 4 to 14 weeks and all but one consented to receive health-related secondary findings). See Table 4 for illustrative quotes.

**Table 4:** Understanding and attitudes towards secondary findings.

Categories	Themes	Example quotes
Understanding	Cancer	*“they said they looked at cancer” P03*
	Heart disease	*“heart disease for instance” P07*
	Misunderstandings about results types	*“I think it was Huntington’s” P16*
	Misunderstandings about level of control	*“I think there was a comment box ‘is there anything else you would be interested in knowing?” P13*
	Unable to recall being asked	*“If I was asked that I honestly can’t remember.” P21*
	Unable to recall decision	*“I don’t really remember but I would have thought we would have ticked the box to say that we was happy for any further investigations to be done.” P14*
Perceived benefits	Preparedness	*“forewarned is forearmed as far as I’m concerned” P01*
	Influence lifestyle	*“if it does come back [as increased risk] try and change your diet or do some more exercise” P11*
	Information valuable due to role as a carer	*“Jake is going to need care going forward, so actually my health and wellbeing is incredibly importance for this situation.” P01*
	Family history	*“My wife she has some family history of breast cancer, so I mean if something comes up on that as part of this study then that is something I suppose we’re interested in” P13*
	Information seekers	*“I’d rather be given everything and know what’s going on and then deal with it as and when it happens” P05*
	High-risk result is unlikely	*“it’s most likely not going to happen” P07*
Concerns	Feeling worried or anxious	*“For me personally I think then that’s a whole unknown world where I could possibly be opening up a world of worries for myself, ‘or what about this or what about that?’ when in fact those things may never happen.” P13*
	Concerns about implications for life insurance	*“My only hesitation…whether other companies would be interested in the data…as in insurance companies” P15*
	Concerns about future legal changes to protections	*“If I was to take out any new policies there would be an issue if that law was to change, especially for my daughters” P06*

#### Perceived benefits

Almost all participants expressed at least some positive attitudes towards secondary findings, with around half expressed only positive attitudes. Many commented that if they were at increased risk they could be more prepared to *“do something about it, prevent it”*, with participants using terms referring to empowerment, such as *“forewarned is forearmed”* when talking about the potential benefits. A few commented that being at increased risk might influence lifestyle e.g. changing diet or doing more exercise; others reflected on their specific role as carer of a child with a rare disease as a main motivation for consenting to secondary findings. Several referred to their own family history of conditions such as cancer and heart disease, and indicated that they valued the opportunity to know more about whether those conditions might be passed on. Several referred to the type of person they were, describing themselves as information seekers who *“wants to know everything”* and another as *“fairly laid back”*. Some of those participants were unconcerned about secondary findings because they perceived a high-risk result to be unlikely e.g. one participant explicitly said that he thought his risk was likely to be low.

#### Concerns

Although many participants expressed only positive attitudes towards receiving secondary findings, just under half expressed both positive and negative attitudes; in addition, as noted above, a few couldn’t recall the conversation, or felt they did not receive enough information to form a view one way or another. Thus, whilst the decision to opt-in to secondary findings appeared clear-cut for some participants, around a third cited both benefits and harms indicating they were conflicted, ambivalent and found the decision *“tricky.”* The main concern about secondary findings was concern about the potential emotional impact of results, and feeling worried or anxious about receiving secondary findings. Some people extrapolated from other areas of their life when thinking about the secondary findings, e.g. one reflected on the experience when his son had been critically ill, commenting; *“more often than not it helped to know less…I protected myself in terms of getting information overload and then worrying about the future.”*

A few participants had concerns about implications of secondary findings for life insurance. A couple of the participants spoke about the re-review of the moratorium on genetics and insurance that is taking place in the UK in 2019, and how this had been an issue they had raised with the consenter, in particular questioning whether and how it might affect their current life insurance policy, and expressing concern that the law might change in the future, e.g.

*“If I was to take out any new policies there would be an issue if that law was to change, especially for my daughters”* (P06)

Similarly, another participant was still unclear following the consent appointment as to whether a change in the law would affect her life insurance, but commented that, *“even if we had to tell them in 2019…I don’t think I’d withdraw from the study.”* Two other participants had had their initial concerns about insurance put *“to rest”* by the consenter. One of these said he had been reassured about insurance by the consenter telling them that they *“didn’t have to pass [the information] on if [they] don’t want to”*.

### Decision support

Overall, participants were very positive about the consent appointments, describing them as *“cohesive,” “informative,”* and *“in-depth”*. However, a few commented about the amount of information conveyed; in particular, several said that having the discussion about the secondary findings as well as the main findings and research aspects in the same appointment was *“bamboozling”* and *“a lot to take in and a lot to process”*. One participant wondered whether *“this conversation about additional findings [was] a bit too close on the back of the other one.”*

Feedback about the information sheets was mixed. A few had not read them, commenting that they did not have time as their life was too *“hectic”*. One said this wasn’t an issue as any questions she had could be addressed during the appointment. Whilst some had positive comments such as *“informative”, “straight to the point”* and *“easy to understand”*, others felt there was too much information in the leaflet, and one mother felt that the focus had been on her child taking part in research rather than getting him a diagnosis which had made her *“seriously consider not doing it”*. Importantly, this latter participant talked at length about how important the conversation with the consenter had been in helping her understand that getting a diagnosis for her son was actually an important focus for the project, and that on the basis of this conversation she had subsequently decided to take part.

A number of participants said additional sources of support e.g. online educational tools would have been valuable alongside the consent appointment. Their suggestions included *“animations”* or *“videos”*, as well as static visual information such as *“pictures”* and *“simple diagrams,”* rather than only text-heavy paper-based information sheets. Online tools were considered particularly useful because they can be shown *“in steps,”* because people can *“go back to it at any point,”* and because they may be particularly useful for young children and those with learning difficulties.

When asked what other questions the information sheet could address, responses included what additional findings would be looked for, more information on how genome sequencing is done, and whether other family members will receive sequencing results without the knowledge of the primary carer. As noted above, one participant strongly felt the information sheet should also have included far greater emphasis on the diagnostic purpose of the sequencing, as against primarily the data and research uses. Another participant said it should be made clearer that parents would also be having their genome sequenced. A final suggestion was that the information sheet should have made it clearer that they would be asked to make a decision about secondary findings at the consent appointment.

## Discussion

In addition to providing clinical diagnoses for families and data for discovery research, the 100,000 Genomes Project provides an important opportunity to learn from patients themselves about their experiences and outcomes. In this qualitative interview study, we have identified a broad range of themes that emerge when participants talked about their attitudes and understanding, which we have drawn on to inform the development of new quantitative measures assessing attitudes and knowledge for larger-scale research (manuscript in preparation). Furthermore, we identified several key themes that may have particular relevance to education, training and policy going forwards.

### Attitudes towards data being used for research

Parents taking part in the 100,000 Genomes Project were understandably motivated by a desire to obtain a diagnosis, but were also very positive about helping others and contributing to research, findings that support previous research in this area [20, 21]. This is supportive of the ‘social contract’ as described in the UK Chief Medical Officer’s annual report Generation Genome [23] and in reports on patient [24] and public views [25] regarding genomic medicine commissioned by Genomics England.

However, although participants were very positive about the research aspects of the project including data sharing, a novel finding from this work was that some participants still valued the in-person consent conversation because it gave them reassurance around data security and protection. This suggests that written information materials—even if read—may perhaps not always be able to give adequate reassurance on this issue. Authors of a recent report on public attitudes to commercial access to health data found that participants would have been more trusting if they had known more about the processes and safeguards in place around data collection and sharing [26]. Our findings reinforce the importance of providing patients with the opportunity to ask questions in addition to providing written information materials at the time of consent. These findings could be useful for training of health professionals receiving consent and discussing the decision with patients. Even if consent conversations in future clinical contexts are very brief, training about what type of data security questions patients have e.g where is data stored, how it is anonymised, who has access to it, how and by whom is access granted etc will help clinicians talk to and reassure patients about how their personal data will be protected when used for research. This could include animations explaining how data is protected, such as those developed by Genomics England for the 100,000 Genomes Project, could also be adapted for use in the new NHS Genomic Medicine Service [27].

#### Issues around secondary findings

Participants raised several issues around secondary findings. This included misunderstandings about what types of conditions were included on the list of secondary findings (participants were informed they would learn about clinically ‘actionable’ findings, which was the term used in the consent form, however, we have observed that consenters did frequently cite ‘cancer’ and ‘heart disease’ during consent appointments [28]), as well as whether they had personally opted to receive to secondary findings. These findings are consistent with a previous UK study in which many participants couldn’t recall their decision about secondary findings at the time of consent [15]. Where our findings add to the existing research is that we also found that several parents felt it was overwhelming to have the conversation about secondary findings at the same time as the conversation about the main findings. Whilst this is a concern that has been raised elsewhere in the literature [29], we are unaware of other studies where this concern was realised in clinical practice. In our previous study in which 21 consent conversations between consenters and potential participants in the 100,000 Genomes Project were audio-recorded and analysed, we found that in most cases consenters spent relatively little time (usually <5 min) discussing secondary findings with potential participants [28]. Taking more time to discuss secondary findings may alleviate the risk of patients being overwhelmed by information. However, a tension exists between the need to provide comprehensive pre-test counselling, including allocating time for patients to consider their decision, and at the same time the practical reality of time-constrained appointments.

Together, our findings have implications for policy and practice as genome sequencing starts to be integrated into clinical practice in the UK and elsewhere globally. If the option of receiving secondary findings is offered at the same time as consent for main findings, greater time for the secondary findings discussion is needed. This may incur additional costs and resources and online decision support tools that complement the in-person conversation may therefore be particularly useful in this context. A number of web-based decision aids have been developed to support patients making genomic testing decisions, reduce decisional conflict and enhance the patient-provider communication in recent years [30, 31]. The Genomics ADvISER was specifically developed to support patients’ decisions about learning secondary findings [30] and is current being evaluated through a mixed methods randomised controlled trial [32].

However, participants’ main concern about secondary findings was the potential psychological harm that the results might have on them. This concern has been identified in previous qualitative research [12, 15]. In our previous observational study [28], most of the consent conversation was dedicated to providing patients with biomedical information and information about the project, e.g. data security, rather than discussing potential psychosocial impact. A US group recently recommended that ‘complex’ findings with potential psychological and serious health implications such as *BRCA1/2* should involve a dedicated conversation with an appropriately trained healthcare professional and with appropriate supporting information materials [33]. While this may not be realistic going forwards, if secondary findings (including *BRCA1/*2) are to be returned in future clinical settings, empirical evidence is urgently needed to inform best practice and policy decisions about consent and pre-test counselling, education and the need for follow up appointments. This includes evidence on both the patient-provider communication around the time of consent, and the psychological impact of secondary findings once they have been returned.

Concerns about implications of secondary findings for life insurance were also raised and not easily addressed because of possible future changes to legal and other protections. The finding that people considering genetic or genomic testing are concerned about insurance is not a new observation [15, 34]. However, our study adds insight on people’s views regarding protections in this area. Patients and research participants are often informed that they are protected in terms of insurance, e.g. GINA in the US [35], and the Code on genetic testing and insurance in the UK [36]. However, our findings indicate that at least some people are not reassured by these current protections, because they recognise these might be removed or changed in the future. Training for clinicians offering genomic testing to patients therefore needs to take this into account. Clinicians need to be prepared to answer patients’ questions about the implications of genomic testing for life or health insurance; they need to be up-to-date on current policy on protections and foreseeable changes to these where possible, or at least know where (e.g. relevant websites) to direct patients/families to find out more.

Limitations of our study include that it was a small qualitative study and so may not be generalisable to the population from which the sample was drawn. In addition, all the interviews were done over the telephone, which may have contributed to the brevity of some of the interviews. Unfortunately, due to repeated rearrangement of interviews in a number of cases, there was a large time-lag between the consent appointment and the interview taking place (in one case this was 17 weeks). This may have impacted recall of information given about the study. Interviews were conducted before the parents had received any main or secondary findings and their views may change after receiving these findings. Thus evaluation of attitudes after return of results is an important line of investigation for future research. It is also important to bear in mind that going forward, genome sequencing will be used as a first-line test for many rare disease patients, and unlike the participants in the 100,000 Genomes Project, these patients may not have had any previous genetic testing. Their support and information needs may differ to the participants in the 100,000 Genomes Project; for example they may less familiar with genetic and genomic concepts or the limitations and uncertainties associated with this type of testing. However, our findings provide novel insights into UK parents’ current attitudes, concerns, understanding and information needs, provides empirical evidence for policymakers, and are of value to researchers developing interventions for patients undergoing genomic sequencing.

In conclusion, our study findings suggest that current consent procedures may effectively support parents in decision-making about genome sequencing for diagnosis and research, but also that more research is warranted into development and evaluation of online decision support tools, and whether and how secondary findings from genome sequencing should be offered clinically. Additional qualitative as well as large-scale research on outcomes after personal results have been returned to parents and patients will also be vital to inform the development of policy and practice.

## Supplementary information

Supplementary Material
