# Supplementary material for: Parents’ motivations, concerns and understanding of genome sequencing: a qualitative interview study
Source: Eur J Hum Genet. 2020 Jan 30;28(7):874–84. doi: 10.1038/s41431-020-0575-2 (PMC7316711; doi:10.1038/s41431-020-0575-2)
Supplement: Supplementary file 1 — Supplementary Material [file 41431_2020_575_MOESM1_ESM.docx]

**INFORMED CHOICE ABOUT WHOLE GENOME SEQUENCING**

**IN-DEPTH INTERVIEWS WITH PATIENTS / RELATIVES OF PATIENTS WITH A RARE DISEASE**

**INTERVIEW TOPIC GUIDE**

**OBJECTIVES**

1. To explore **understanding** of WGS (including **recall** and **expectations**)
2. To explore **attitudes** towards WGS (including **values, benefits** and **concerns**)To explore **motivations** for accepting or declining WGS
3. To explore thoughts, feelings and decision-making about the **primary results**
4. To explore thoughts, feelings and decision-making about the **secondary results**
5. To explore **information needs** regarding making an informed choice
6. To explore **satisfaction** with informed consent documents and procedures
7. To gather reflections on how the WGS consent procedure could be **improved**

**[Introductory Script]**

Hi. My name is ______________ (first name) and I’m from Great Ormond Street Hospital. I’m calling you because you very kindly agreed to do the interview to learn about your thoughts and feelings about having your genome sequenced as part of the 100,000 Genomes Project. I’m going to be doing the interview with you. Is this a good time to talk?

*If no:* OK no problem, when would be convenient for me to call you back?

*If yes:* OK great.

As you know, we are planning to tape record this phonecall so I can listen instead of taking notes, and so that we make sure we understand everything you say correctly. Is that ok?

*If yes: proceed with interview.* Wonderful. I’m going to be asking you a number of questions about when you were invited to take part in the 100,000 Genomes Project, your thoughts and feelings about doing this, what your expectations are, if there was anything that could have been done differently or done better in the future. Does that sound OK?

Before we start, did you have a chance to read the information sheet about the study that we sent you? Do you have any questions about the information sheet you were sent or anything else relating to this study?

Before we start, I just want to read through the consent form. **[initial boxes if yes to each; sign on behalf saying ‘telephone consent’; sign for yourself as researcher]**

***Then proceed with interview***

1. **Understanding, recall & expectations**

- First, would you tell me a little bit about you and your family, why you were invited to participate in the 100,000 Genomes Project?
- In their own words, what happened when they were invited to participate in the 100,000 Genomes Project
  - How / when did they first hear about the project
  - What happened during the informed consent procedure
  - Had they ever had any other kinds of genetic tests done before they were invited to take part in the 100,000 Genomes Project?
  - What are the key points they remember discussing with the health professional before they made their decision about whether or not to have whole genome sequencing done (or consent for their child to have whole genome sequencing done)?
- Probe understanding of whole genome sequencing
  - What are their expectations around receiving test results
  - What are their expectations of what they will find out?
  - What is their understanding of:
    - How their genomic data will be used
    - Where their genomic data will be kept
    - Who has access to their genomic data
    - How long analysis will take
    - Process for withdrawing consent if they change their mind
  - Any other details

1. **Attitudes**

- What are their overall thoughts about having whole genome sequencing?
- What do their think are the benefits?
- What are their concerns?
- How are they currently feeling about your decision to have genome sequencing done?
  - Primary results
  - Additional findings (disease, carrier)

1. **Motivations**

- What were their reasons for accepting WGS / declining WGS?
- Did they opt to participate? Why / why not?
- Did they opt to receive the additional disease findings? Why / why not?
- Did they opt to receive the additional carrier findings? Why / why not?

1. **Decision: primary findings**

- Explore their thoughts, feelings and decision-making specifically about the primary results (i.e. diagnostic results relating to their / their child’s rare disease

1. **Decision: secondary findings**

- Did they decide to find out about secondary, additional findings relating to disease risk
- What were their reasons were for deciding to receive / not receive additional disease findings?
- Explore their thoughts, feelings and decision-making specifically about the primary findings (i.e. result relating to their / their child’s rare disease)

1. **Information needs**

- What information was it most important for them to know when they were making their decision about having genome sequencing done?
- What are the most important things that they think should be included in patient information sheets about WGS?
- One of the things we’re interested in is whether people are making what are sometimes called “informed decisions” when it comes to having genome sequencing done. What does an informed decision mean to you?
- Do you feel you made an informed decision about having genome sequencing done?

1. **Satisfaction**

- Explore satisfaction with procedures and materials
  - Were there any aspects of the discussion with the health professional they found confusing or unclear?
  - Were there any aspects that they felt were really helpful for their understanding? Looking back, is there anything they wish they had asked that they didn’t ask at the time?
  - What did they think of the information sheet and informed consent form?
  - Was there any further information they would have liked, but which they did not receive?
  - Would they have preferred to receive the information in another format (e.g. video)?
  - What are their views of the consent form that they signed?
  - Did the form cover what they think it was important to cover with regard to consent?
  - Was the consent form clear?

1. **Improving procedures and documents**

- Do they feel they had enough time to make their decision about having whole genome sequencing done?
- If not, how much time would it have been better to have had before making this decision?
- Is there anything they think can be improved about the way health professionals discuss whole genome sequencing? What do they think is the best way to inform patients / parents about whole genome sequencing? What do they think could be improved?

1. **Additional comments**

Any other comments, suggestions, feedback they would like to give regarding protocol, communication, consent counseling, impact on them, any other aspect of study

***When finished: Ask if happy to be contacted again, if they have any questions, and thank them for their time.***
